# Better mealtimes for people living with dementia: Results from a feasibility study

**DOI:** 10.1002/alz70858_099233

**Published:** 2025-12-25

**Authors:** James Faraday, Andrew McCarthy, Peter Van der Graaf, Annette Hand

**Affiliations:** ^1^ The Newcastle upon Tyne Hospitals NHS Foundation Trust, Newcastle upon Tyne, United Kingdom; ^2^ Newcastle University, Newcastle upon Tyne, United Kingdom; ^3^ Northumbria University, Newcastle, United Kingdom; ^4^ Northumbria University, Newcastle upon Tyne, Tyne and Wear, United Kingdom; ^5^ Northumbria University, Newcastle, Tyne and Wear, United Kingdom; ^6^ The Newcastle upon Tyne Hospitals NHS Foundation Trust, Newcastle upon Tyne, Tyne and Wear, United Kingdom

## Abstract

**Background:**

Mealtimes are important for health and well‐being. Some people living with dementia experience challenges at mealtimes, including problems using cutlery, reduced range of tastes and preferences, and difficulties with swallowing. In nursing homes, care staff provide direct care at mealtimes, but do not consistently receive high‐quality training on this topic. This feasibility study investigated the acceptability of an evidence‐based training programme which promotes good practice in mealtime care for people with dementia.

**Method:**

The training was delivered in‐person in three nursing homes in the UK. The homes were selected to be different in certain key attributes (such as number of beds, and type of ownership). Staff members from across all departments in the homes were eligible to participate. Before and after the training, participants completed the Sense of Competence In Dementia care Staff (SCIDS) scale, and a bespoke tool measuring confidence in mealtime care. In addition, focus groups were used to obtain qualitative feedback after the training, and a reflexive diary was used to capture researcher reflections on process and practicalities. These methods were developed in consultation with experts by experience.

**Result:**

Nursing home engagement in the study was facilitated by in‐person meetings with senior staff in each home. Twenty‐nine participants were recruited from a target of 30 (97%), including carers, kitchen staff, and managers. Attendance was high, with 100% of recruited staff attending the training. There was no attrition during the study. All participants took part in the pre‐ and post‐training questionnaires, and the focus groups. A high percentage of participants (89.7%) completed all questionnaire items. Provisional analysis of focus groups data suggests high levels of acceptability for training content and format.

**Conclusion:**

This study has provided useful information to address uncertainties about a future randomised control trial (RCT) of the training programme. In parallel with the feasibility study, a working group including people with lived experience has created an animation which communicates the core messages from the training programme, to disseminate these in an accessible way (https://vimeo.com/1009856313).

